# Pancreaticoduodenectomy on soft-embalmed human cadavers according to Dodge – a pilot feasibility report

**DOI:** 10.1016/j.sopen.2025.05.005

**Published:** 2025-05-21

**Authors:** Dariya Jaeger, Eric Hinrichs, Ralf Schoppe, Gebhard Reiss, Georg Feigl, Benno Mann

**Affiliations:** a Institute of Anatomy and Clinical Morphology, Witten/Herdecke University, Witten, Germany; b MoViDo gGmbH, Essen, Germany; c Augusta Hospital gGmbH Bochum, Department of General, Visceral and Robotic Surgery, Bochum, Germany

**Keywords:** Pancreaticoduodenectomy, Human cadaver, Surgical training, Soft embalming, Dodge embalming

## Abstract

**Objective:**

Pancreaticoduodenectomy (PD) is one of the most complex procedures in abdominal surgery. Nowadays, it is very difficult for novice surgeons to learn the procedure of PD on living patients. New concepts are needed to improve the surgical training of PD, comparable to education in the operating room.

**Method:**

We investigated the feasibility of performing PD on a soft embalmed human cadaver using the Dodge preservation technique, considering all operative steps. Surgery was performed by a certified expert. The settings corresponded to the conditions of the operating room with the original surgical instruments and sutures. Upon completion of the PD, feedback in the form of a comprehensive questionnaire was obtained from the expert by evaluating all relevant operational steps in terms of realism using a 5 point Likert scale.

**Results:**

PD was performed successfully by the expert. The results showed very good feasibility for PD on the used Dodge embalmed cadaver (DeC). The expert confirmed a realistic surgical performance similar to real-life conditions, with good color contrast, clearly visible tissue layers for a layered preparation, and a great result for the reconstruction part of the anastomoses.

**Conclusions:**

New educational methods are needed to improve surgical training of PD. Hands-on training of PD performed on DeC enables a realistic surgical experience and offers a promising educational method for training in pancreatic surgery.

## Highlights

• Pancreaticoduodenectomy is one of the most complex procedures in abdominal surgery.
• New concepts are needed to improve surgical training of pancreaticoduodenectomy.
• Pancreaticoduodenectomy on Dodge's soft-embalmed human cadavers enable a realistic surgical experience.
• Dodge's soft embalmed cadavers offer an excellent opportunity for surgical training of pancreaticoduodenectomy.

## Introduction

Pancreaticoduodenectomy (PD), a complex surgical procedure involving the resection of the pancreas, duodenum, gallbladder, and bile duct, requires extensive training for surgeons to achieve proficiency. Hands-on training methods using human cadavers have been widely recognized as an effective approach for surgical skill acquisition and proficiency development, allowing surgeons to practice and refine their techniques in realistic setting [[1], [2], [3], [4]]. The unique properties of soft embalmed human cadavers, including tissue flexibility and preservation of anatomical structures, could make them particularly suitable for training in complex surgical procedures, such as PD.

Furthermore, the implementation of proficiency-based training programs for specific surgical procedures, including PD, is essential for ensuring safe and effective skill acquisition [5]. These programs aim to provide structured and comprehensive training, enabling surgeons to attain the necessary competence for performing complex surgical interventions [5,6]. These training programs include a proficiency-based virtual reality simulation curriculum, an inanimate biotissue curriculum, video library training, intraoperative evaluation, and skill maintenance with ongoing assessment [6]. However, the utilization of cadaveric workshops in postgraduate surgical training has been shown to have a positive impact on trainees' technical skills performance, further highlighting the effectiveness of hands-on training methods using human cadavers [7].

In summary, hands-on training methods using human cadavers, particularly those embalmed with specific preservation techniques, play a crucial role in the training and skill development of surgeons performing complex surgical procedures, such as PD. The implementation of structured training programs and evaluation of surgical techniques contribute to the continuous improvement of surgical training and practice, ultimately enhancing patient outcomes and safety.

To the best of our knowledge, the literature review did not identify any studies on the feasibility of pancreatic surgery on soft-embalmed human cadavers using Dodge's technique [8].

The objective of this study was twofold: first, to illustrate the viability of a comprehensive PD procedure on a Dodge embalmed cadaver (DeC) by a board-certified pancreatic surgeon as an expert. Second, to examine the extent to which the procedure aligns with the experience of PD procedures on living patients.

## Materials and methods

### Human cadaver - soft embalmed according to Dodge's method

A single human cadaver, embalmed according to Dodge's method, was used. The male human donor was donated to a non-profit prosecture MoViDo gGmbH placed in Essen, Germany, and given to the University of Witten/Herdecke for scientific examination after the embalming process was completed. The human cadaver was used under the strict rule of donation programs and was investigated with the local ethical commission vote of the University of Witten/Herdecke.

Dodge's embalming method using Dodge solutions (Dodge Co., Billerica, MA, USA) is a soft embalming method that creates soft and flexible cadavers with almost lifelike conditions [8]. Furthermore, this embalming method creates a very good color contrast in the tissue. It facilitates dissection and allows the surgeon to work close to the reality of a live patient. Therefore, DeCs can be used in many different clinical fields for scientific investigation and training methods.

### Selection of the human cadaver

No specific selection criteria regarding sex or height were used to select human cadavers. The only requirement was exclusion of laparotomy scars. The cadaver was screened for laparotomy scars by anatomy staff and selected for this study accordingly. No further information was available on the cadavers' previous illnesses or surgeries due to data protection.

### Expert

The surgical procedure was performed by a recognized expert. The expert involved was the director of a surgical clinic and a certified pancreatic surgery center. This expert has proven experience with more than 600 pancreatic resections. In addition, the expert already has extensive previous experience in surgery on human cadavers such as fresh-frozen cadavers, formalin-embalmed cadavers, and Thiel's embalmed cadavers, but no previous experience with Dodge's embalming technique.

### Setting

**Fig. 1:**
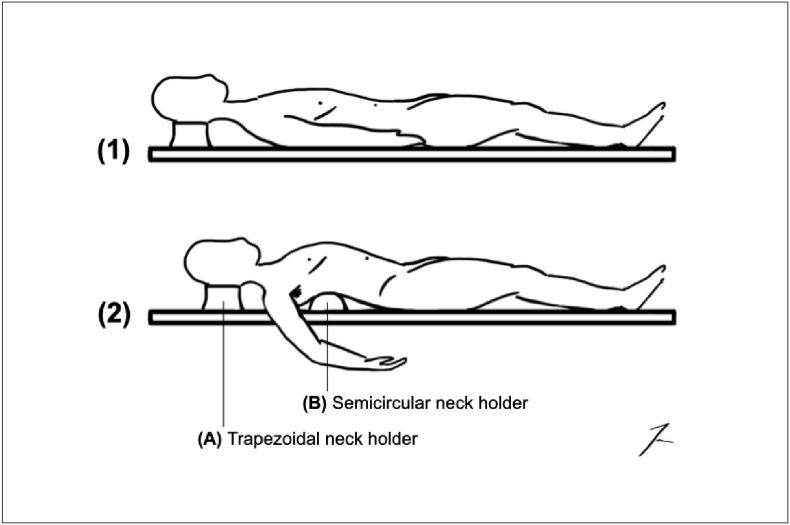
Scheme showing cadaver placement. (1) The cadaver was laid on a non-adjustable preparation table. (2) Cadaver placement using a semicircular neck holder (B) to improve opening of the upper abdomen. The right arm is shown in abduction for a better view of the semicircular neck holder. In (1) and (2), the head is laid on a regular trapezoidal neck holder (A) that can also be used for placement.

The hands-on procedure was performed in the regular dissection room of the Institute of Anatomy. A regular anatomy preparation table was used as the operating table. The table could not be adjusted for height or tilted sideways. In this case, no positioning maneuvers of the cadaver were necessary for the surgical procedure of the PD. If necessary, positioning maneuvers of the cadaver could have been performed manually, for example, by placing a semicircular neck holder underneath to improve the opening of the upper abdomen, as shown in Fig. 1. Above the preparation tables was a fixed powerful lighting system that covered the entire length of the tabletop. Free-standing focusing light was used for deep dissections. For the PD procedure, the surgeon used standard instruments for open surgery, an atraumatic wound protector/retractor, and suture materials. No other surgical equipment was required.

### Surgical technique

**Fig. 2:**
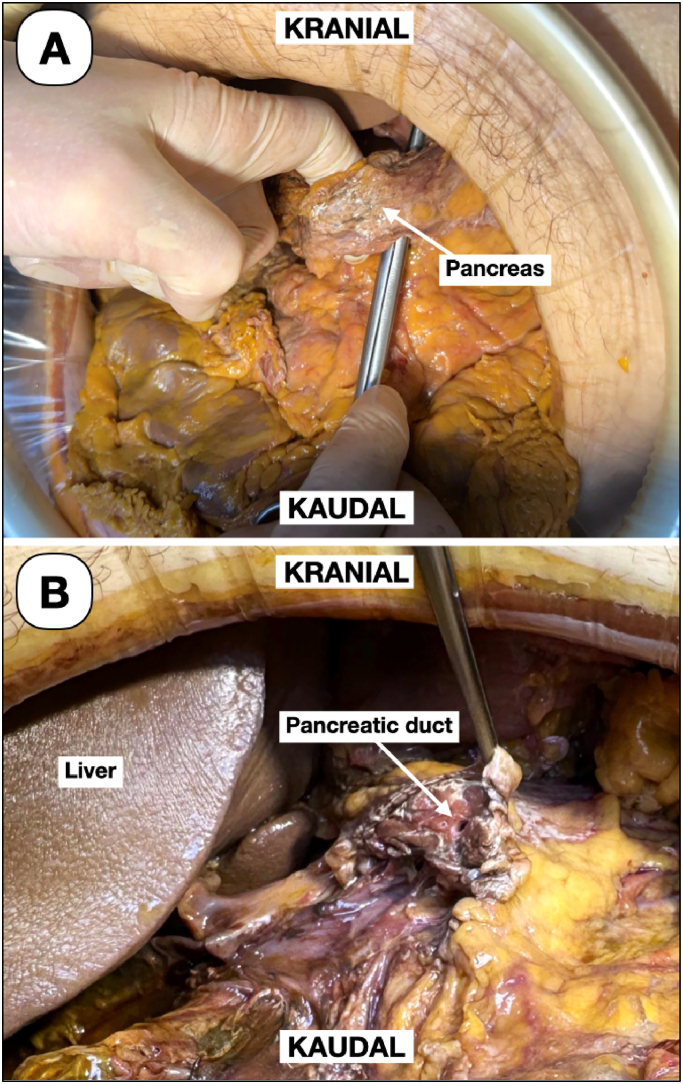
Overview of the upper abdomen in situ of the DeC before reconstruction. (A) Pancreatic mobilisation before pancreatic head resection. (B) The pancreatic duct after resection of the pancreatic head.

The expert performed a standard open surgical, radical oncological pylorus-preserving partial pancreaticoduodenectomy with Blumgart-Style pancreaticojejunostomy [9]. Fig. 2 shows the in-situ overview of the upper abdomen before reconstruction.

### Evaluation questionnaire using Likert scale

The expert was presented with a comprehensive questionnaire for evaluation based on the questionnaire model of Jaeger et al. [1]. The 41 questions were rated on a Likert scale ranging from 1 (strongly disagree) to 5 (strongly agree), as shown in Table 1. The main objective of this study was to analyze the feasibility and realism of cadaveric training compared to surgery on live patients.

**Table 1:** Overview of the questionnaire with expert ratings on a Likert scale (1–5).

X	X – General questions	Expert rating (Likert)
X1	Open surgical, radical oncological partial pancreaticoduodenectomy	
1.	Performing the PD on a soft embalmed human cadaver is a good training for performing the operation on a live patient.	5
2.	Hands-on training of the PD on human cadaver under supervision could make inexperienced surgeons more confident in carrying it out.	5
3.	The procedure on the human cadaver was lifelike.	4
4.	I can recommend hands-on training in the form of a workshop.	5
X2	General questions Preparation of the tissue	
1.	Upon inspection, the color of the tissue is lifelike.	4
2.	Inspection reveals clearly visible tissue layers for a layered preparation.	5
3.	Tissue is soft and elastic.	4
4.	Tissue layers can be easily moved against each other.	5
5.	The preparation of the tissue offers a realistic surgical experience.	4
A	A – Access and exposure of the abdominal cavity Transverse right upper abdominal laparotomy	
1.	The skin incision can be performed realistically.	3
2.	The layered dissection of the abdominal wall can be performed realistically apart from hemostasis.	4
3.	The exploration of the abdominal cavity to rule out peritoneal carcinomatosis and liver metastasis can be performed realistically.	4
B	B – Checking the resectability and removal of the uncinate proc. from the left	
1.	The assessability of the mesenteric root for the exclusion of tumor infiltration was realistically possible.	5
2.	The opening of the omental bursa by splitting the gastrocolic ligament and separating the transverse mesocolon and the mesogastrium in layers was realistically possible.	5
3.	The exclusion of tumor infiltration of the branches of the superior mesenteric artery was realistically possible.	5
4.	The placement of the second jejunal loop approx. 15 cm from Treitz's ligament and the release of the first jejunal loop from the prerenal fascia was realistically possible.	5
5.	The skeletonisation to the superior mesenteric artery and detachment of the uncinate process from the superior mesenteric artery was realistically possible.	5
C	C – Kocher maneuver/resection of the duodenum or distal stomach	
1.	The mobilisation of the duodenum according to Kocher was realistically possible.	5
2.	The mobilisation of the right colonic flexure was possible in a realistic manner.	5
3.	The jejunal loop and the uncinate proc. could be mobilised well into the right upper abdomen.	5
4.	The skeletonisation and removal of the postpyloric duodenum was realistically possible.	5
D	D – Cholecystectomy, lymphadenectomy of the hepatoduodenal ligament, dissection of the hepatic duct	
1.	The antegrade cholecystectomy was realistically possible.	4
2.	The lymphadenectomy of the hepatoduodenal ligament and dissection of the hepatic duct was realistically possible.	4
E	E - Separation of the pancreas/release of the uncinate proc. from the right	
1.	It was possible to drive under and cut through the pancreas realistically.	5
2.	Triggering of the uncinate process from the right was realistically possible.	5
F	F – Reconstruction	
F1	Pancreaticojejunostomy (Blumgart-Style)	
1.	The texture of the pancreas was realistic.	4
2.	The pancreatic anastomosis was realistically possible.	4
3.	The suturing and knotting of the pancreatic anastomosis sutures were realistic.	4
F2	Biliodigestive anastomosis - BDA	
1.	The tissue texture of the hepatic duct was realistic.	4
2.	It was possible to create the BDA realistically.	4
3.	The suturing and knotting of the bile duct anastomosis sutures were realistic.	4
F3	Duodenojejunostomy/Gastrojejunostomy	
1.	The tissue texture of the duodenum and stomach was realistic.	4
2.	The creation of the duodenojejunostomy was realistically possible.	4
3.	The suturing and tying of the duodenojejunostomy was realistic.	4
4.	The creation of the gastrojejunostomy was realistically possible.	Not performed
5.	The suturing and knotting of the gastrojejunostomy was realistic.	Not performed
G	G – Wound closure	
1.	The wound edges can be easily adapted.	5
2.	The needle can be easily guided through the tissue when suturing the wound.	5
3.	The tissue ruptures more in comparison to the living tissue.	2
4.	The knot guidance can be performed realistically.	5
5.	The skin suture on the tissue used offers a realistic suturing experience.	4

The structure of the questionnaire was modular and contained the following parts. General part, including the most important aspects of the workshop and tissue preparation. Following with the anatomical and surgical part with modules on surgical access, dissection and resection part, the reconstruction part, and the wound closure. In addition, specific questions regarding the particularities of cadaver training, such as the ethical aspects of surgical training on human cadavers, the absence of hemorrhage, and the lack of time pressure with its training effects. All questions were aimed at analyzing the realism of cadaver training compared to surgery on patients. An overview of the questionnaire and the results of the expert rating are highlighted in Table 1.

## Results

### General

All general questions were answered with a Likert score of 4 (agree) or 5 (strongly agree). The expert strongly agreed that performing PD on DeC is a good training for performing the operation on a live patient, which could make inexperienced surgeons more confident in carrying it out. Tissue preparation was perceived to show a realistic surgical experience with a median score of 4, showing clearly visible layers for a layered preparation.

### Procedure of the PD

Surgical access with transverse right upper abdominal laparotomy and exploration of the abdominal cavity were rated with a median score of 4 (agree). Checking the resectability and removal of the uncinate processus from the left as well as the Kocher maneuver were perceived as lifelike with a median score of 5 (strongly agree). Cholecystectomy, lymphadenectomy of the hepatoduodenal ligament, and dissection of the hepatic duct were perceived as lifelike, with a median score of 4 (agree). The reconstruction part of all anastomoses (pancreaticojejunostomy (Blumgart-Style), biliodigestive anastomosis, duodenojejunostomy) was perceived as lifelike with a median score of 4 (agree), including the tissue structures and a realistically feasible suturing and knotting of the anastomoses. In the present study, pylorus-preserving PD with corresponding duodenojejunostomy reconstruction was performed. Consequently, questions 4 and 5 in module F3 on gastrojejunostomy reconstruction were marked as “not performed”.

Wound closure (module G) was also perceived as very lifelike, with a median of 5 (strongly agree). It is notable that question G3, which states, “The tissue ruptures more in comparison to the living tissue,” was answered with a rating of 2 (disagree), indicating that the expert did not believe that the tissue tends to rupture more than the living tissue.

**Fig. 3:**
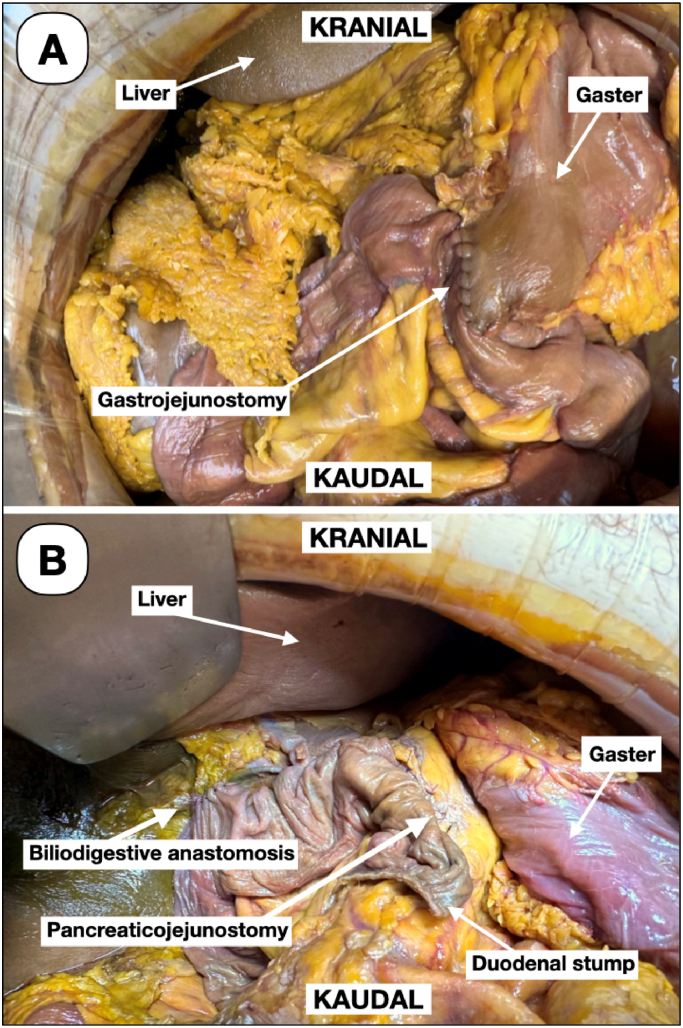
Overview of the upper abdomen in situ of the DeC after reconstruction. (A) Gastrojejunostomy. (B) Pancreaticojejunostomy and biliodigestive anastomosis.

Fig. 3 shows the in situ overview of the upper abdomen after reconstruction.

## Discussion

PD is one of the most complex surgical procedures in abdominal surgery and requires intensive training of the young surgeons.

The comparison of different surgical techniques and the assessment of factors predictive of surgical outcomes following PD have been subjects of extensive research, reflecting the ongoing efforts to enhance training and optimize surgical practices in this field [[10], [11], [12]]. Moreover, the implementation of proficiency-based training programs for complex surgical procedures, including PD, has been identified as a crucial element in ensuring safe and effective acquisition of skills [5]. However, the effectiveness of training on human cadavers has not yet been studied. Our search identified a single previously published paper in which perfused fresh cadavers, which had been neither embalmed nor frozen, were utilized for training purposes in robotic minimally invasive pancreaticoduodenectomy (MIPD) [13]. These authors demonstrated that perfused fresh cadavers represent a safe and effective method for MIPD training with a highly realistic dissection and the opportunity to develop and practice essential technical skills for these complex procedures. In addition, the effectiveness of training programs that include virtual reality training and bio-tissue exercises has been demonstrated, but it has also been noted that these techniques do not fully simulate the intraoperative experience and tissue handling [13].

Cadaver training is a promising method to learn and train the complex surgical procedure of PD. However, the particularly fragile parenchyma of the pancreas poses a difficulty. Various pre- and post-mortem factors can lead to autodigestion of the pancreas and destruction of the parenchyma, which, in turn, can impair realistic training. The various influencing factors remain to be investigated. Therefore, the condition of the pancreas before cadaver preservation is difficult to assess without exploration by open surgery or laparoscopy, regardless of the preservation method used postmortem.

Nevertheless, the preservation solution appears to play a major role in preserving the pancreatic parenchyma and its original consistency. A pilot study of a hands-on workshop on soft-preserved cadavers according to Thiel's method was conducted at the University of Witten/Herdecke in 2024, in which trainees performed a complex surgical procedure of open-surgical radical cystectomy on five cadavers under the supervision of experts [1]. The results of the trainees' training experience showed a higher effectiveness of surgical education compared to teaching at the operating table and confirmed a high degree of realism in the training of open radical cystectomy and urinary diversion on human cadavers in soft-embalming according to Thiel's method. In our experience, the pancreas was regularly extremely soft, necrotic, and/or autolytic when using the well-studied soft preservation method, according to Thiel [14]. To our opinion, it can be assumed that the highly fat-soluble preservative solution of Thiel's method may affect sensitive pancreatic parenchyma. The same phenomenon can be observed in almost all glandular tissues with Thiel's soft-embalming, for example, in the thyroid gland, and negatively affects the preservation of the central nervous system (cerebrum and spinal cord). With DeCs, on the other hand, we observed good results in the preservation of sensitive organs such as the cerebrum and spinal cord, so we assumed that the pancreas could also be well preserved.

To our knowledge, no studies on the feasibility of pancreatic surgery on DeCs could be found in the literature search to date. In this study, we demonstrated the feasibility of PD on DeC. Using a comprehensive questionnaire, a recognized expert was able to assess the feasibility and high realism of the complete open PD procedure on a DeC, with remarkable results. However, this assessment was based on the experience of a single expert on a single cadaver. Further follow-up studies are needed to include more experts and investigate the transferability of the procedure to different cadavers. The impressions of novice surgeons as mentees would be at least as important as the expert opinion of the supervising mentors and should be included as well. Furthermore, it would also be useful to investigate the specific features of surgical training on human cadavers, such as ethical aspects and the advantages and disadvantages of the lack of blood flow.

While this study investigated open PD, the outlook is also adaptable to laparoscopic and robotic PD. In the context of PD, the significance of training programs for laparoscopic and robotic approaches has been underscored, with studies emphasizing the need for specialized training to facilitate safe implementation of these advanced techniques [5,15]. The transferability of DeC cadaver training for PD for laparoscopic and robot-assisted surgeries should be investigated.

## Conclusions

In conclusion, it was possible to perform the complete PD procedure on DeC with excellent results in terms of realism compared to living patients. Surgical training of PD on DeCs is, therefore, a promising possibility to realistically train this complex surgical procedure. Further studies on the transferability of the concept in workshop format are underway.

## CRediT authorship contribution statement

**Dariya Jaeger:** Conceptualization, Data curation, Formal analysis, Investigation, Methodology, Project administration, Resources, Software, Validation, Visualization, Writing – original draft, Writing – review & editing. **Eric Hinrichs:** Formal analysis, Writing – review & editing. **Ralf Schoppe:** Conceptualization, Resources, Writing – review & editing. **Gebhard Reiss:** Conceptualization, Formal analysis, Methodology, Supervision, Writing – review & editing. **Georg Feigl:** Conceptualization, Formal analysis, Methodology, Supervision, Writing – review & editing. **Benno Mann:** Conceptualization, Data curation, Formal analysis, Methodology, Resources, Supervision, Validation, Writing – original draft, Writing – review & editing.

## Ethics approval

This study was performed in line with the principles of the Declaration of Helsinki. Approval was granted by the Ethics Committee of the University of Witten/Herdecke (21.10.2024/No. S-279/2024).

## Declaration of competing interest

The authors declare that no funds, grants, or other support were received during the preparation of this manuscript.

The authors have no relevant financial or non-financial interests to disclose.
